# Somatic healthcare utilisation patterns among older people with intellectual disability: an 11-year register study

**DOI:** 10.1186/s12913-016-1880-x

**Published:** 2016-11-09

**Authors:** Magnus Sandberg, Gerd Ahlström, Anna Axmon, Jimmie Kristensson

**Affiliations:** 1 Department of Health Sciences, Faculty of Medicine, Lund University, P.O. Box 157, SE-221 00 Lund, Sweden; 2 Department of Occupational and Environmental Medicine, Lund University, SE-221 85 Lund, Sweden; 3 Department of Health, Blekinge Institute of Technology, SE-371 45 Karlskrona, Sweden

**Keywords:** Aged, Aging, Healthcare utilisation, Inpatient, Intellectual disability, Outpatient, Register studies, Retrospective studies

## Abstract

**Background:**

People with intellectual disabilities (ID) are known to have more diseases and are believed to start aging earlier than the general population. The population of older people with ID is growing, but knowledge about their use of healthcare is limited. This study aimed to explore somatic healthcare utilisation patterns among people with ID living in Sweden, in comparison with the general population from 2002 to 2012.

**Methods:**

Participants were a group of people with ID (*n* = 7936) aged 55 years and older in 2012, and an equal-sized, birth year and sex matched, general population sample (*n* = 7936). Participants were divided into age groups of 5-year intervals. Data regarding in- and outpatient care were collected from the Swedish National Patient Register.

**Results:**

In the younger age groups, the ID group had higher healthcare utilisation compared with the general population sample, with higher risks for planned and unplanned somatic in- and outpatient care, particularly for unplanned inpatient registrations. Decreasing patterns were seen with age; with lower risks in the ID group for the oldest age groups. This was most evident in planned somatic in- and outpatient care. In those with at least one registration, the ID group had a longer unplanned length of stay in the younger age groups, but fewer planned visits to physicians in somatic outpatient care compared with the control group.

**Conclusions:**

Compared with the general population, people with ID show higher healthcare utilisation in younger age groups. Healthcare utilisation decreases with age, and in old age, fewer people with ID use healthcare compared with the general population. The barriers to accessing planned healthcare for older people with ID need more investigation.

**Electronic supplementary material:**

The online version of this article (doi:10.1186/s12913-016-1880-x) contains supplementary material, which is available to authorized users.

## Background

Knowledge about healthcare needs, healthcare utilisation and access to healthcare among older people with intellectual disabilities (ID) is sparse. The number of older people with ID is increasing rapidly [1] and they experience high rates of multi-morbidity [2, 3]. Therefore, it is important to gain a deeper understanding about the healthcare needs of people aging with ID, and the health system’s ability and readiness to meet these needs.

People with ID differ in terms of overall health compared with the general population, and have a higher prevalence of chronic conditions such as diabetes mellitus [4], cardiovascular diseases [5], osteoporosis [6], visual and hearing impairments [7, 8], epilepsy, musculoskeletal diseases [8], and thyroid conditions [7]. They also experience high rates of health risk factors such as obesity [9, 10], physical inactivity, sedentary lifestyles, and inadequate emotional support [4], and are reported to have high, or undetected, healthcare needs [8, 11–15]. However, as only one of these previous studies [9] focused on older people, more research is needed on this topic.

The aging process is believed to start earlier in people with ID, which causes earlier onset of age-related diseases [7, 13, 16, 17] and a corresponding increase in healthcare needs. The World Health Organization has expressed concern that the increasing proportion of older people in general means that limited healthcare resources will be primarily used to satisfy general population needs, and only secondarily for the needs of people with ID [18]. This is problematic, as people with ID are already disadvantaged in accessing healthcare and social services [18]. Therefore, studies that compare the utilisation of healthcare in older people with ID with that of the general population are important.

Various forms of healthcare utilisation in people with ID have been internationally investigated; for example, hospital admissions, use of social services, primary care, outpatient specialist care, and rehabilitation services [15, 19–40 41, 42]. However, most of these studies have limitations in terms of determining casual relationships, such as being cross-sectional [15, 19–32, 34–38], focusing on only one type of utilisation [15, 20–23, 25, 27–32, 35, 37, 41], small sample size [23, 24, 27, 33, 37], or they included people of younger ages [21, 22, 28–35, 40]. Therefore, the current evidence base is weak. To our knowledge, only one study focusing on healthcare utilisation across time in people aging with ID has been conducted [41].

Previous research has shown that 10–19 % of people with ID are admitted to hospital each year [26, 30, 33, 34, 36, 41, 42]. The average length of stay (LOS) is reported to be 3.7–6.7 days [19, 28, 33, 41], although one study found a mean LOS of 16.9 days [28]. However, only two of these studies were longitudinal [33, 41], and only three made comparisons with a general population sample or official general population statistics [19, 41, 42]. Studies investigating outpatient care have reported that 82–92 % of those with ID had at least one outpatient/general practitioner visit in the past 12 months [15, 34] and had an average of 1.3–5.4 annual contacts [15, 23, 37].

In summary, there are few available studies investigating inpatient and outpatient healthcare utilisation among older people with ID that have a large sample, a longitudinal design, and a matched control group. Such studies are important to detect changes over time, make valid interpretations of healthcare utilisation patterns, and determine if these patterns differ between people with ID and the general population. Most previous studies also investigated one specific form of healthcare utilisation. However, as people with ID may have their health needs met in various parts of the health system, a more comprehensive investigation is needed that covers both inpatient and outpatient care.

Therefore, this study aimed to explore somatic healthcare utilisation patterns among people with ID living in Sweden compared with the general population from 2002 to 2012.

## Methods

### Study design

This was a longitudinal, retrospective, population register-based study that included people with ID and a birth year- and sex-matched general population sample.

### Setting

The population in Sweden is about nine million, and about 18 % of them are aged 65 years or older [43]. Sweden has also one of the ten highest average life expectancies in the world with an average life expectancy of almost 84 years for women and just below 80 years for men in 2011 [44].

Sweden has a welfare system mainly funded by taxes and thus, the Swedish population is covered by a national health insurance and by this, have equal access to healthcare [45]. Because of this private health insurances are less common in Sweden, with only four percent that have such an insurance. These are also mostly common within occupational healthcare services and are therefore in most cases paid for by employers [45]. Healthcare and social services are regulated by three laws: the Health and Medical Services Act [46], the Act concerning Support and Service for Persons with Certain Functional Impairments [47], and the Social Services Act [48]. Healthcare and social services are provided by municipalities and county councils at different levels. In Sweden there are 21 county councils that are responsible at a regional level for providing healthcare services to their populations (including specialized medical care in outpatient and inpatient facilities, rehabilitation and home nursing care) [45]. The 290 municipalities provide local-level healthcare and social services and is responsible to meet the care and housing needs of older people and people with disabilities. Care is provided in either the person’s own home or in special accommodation/group homes. All healthcare and social services provided within these three laws by the county councils and muncipalities are recorded in mandatory public national registers.

Provision of inpatient and outpatient specialist care related to the Health and Medical Services Act [46] is registered in theSwedish National Board of Health and Welfare’s National Patient Register (NPR). The purpose of this patient registry is to follow health trends in the population, improve the prevention and treatment of diseases, and contribute to the development of healthcare. The registry provides statistics from 1964 for the evaluation of healthcare and research.

Support and services provided under the Act concerning Support and Service for Persons with Certain Functional Impairments [47] are recorded in the National Board of Health and Welfare’s LSS-register. One part of this register is a three-group classification, or personae, that classify the impairment of the service user. Persona 1 represents people who have ID from birth or an early age, with autism or conditions similar to autism; persona 2 represents people who have considerable and permanent mental impairment following brain damage as an adult; and persona 3 represents people who as a result of other serious and permanent functional disabilities, which are clearly not the result of normal ageing, have considerable difficulties in everyday life and great need of support or service [47].

### Study populations

The ID group comprised all people who received support and social services according to the Act concerning Support and Service for Persons with Certain Functional Impairments [47] during 2012, which were registered as persona 1, and who by the end of that year were aged 55 years or older. It is believed that the aging process is likely to start earlier in people with ID [18], but how much earlier is not known and most likely varies with type of ID. We chose a threshold of 55 as it seemed reasonable in relation to this and to the definition of old used for the general population, i.e., 65 years of age. The ID population was identified through the national LSS-register.

A randomly selected control group comprising people from the general population, matched by birth year and sex, was identified from the Swedish National Population Register [49]. We did not match on socioeconomic variables such as income level or educational level. These may be parts of the casual chain between ID and healthcare utilisation. The matching procedure was carried out by Statistics Sweden [50]. The ID group comprised 7936 individuals, and with the equally large control group the study in total included 15,872 participants. A person that had been included in the ID group could not also be selected as a control.

### Material

Anonymized in- and outpatient data were collected from the NPR for the 11-year period from January 1, 2002, to December 31, 2012. This included information about inpatient care (e.g., hospital/clinic, date of registration, whether or not the admission was planned, and LOS) and outpatient care (e.g., hospital/clinic, date of physician visit and whether or not the visit was planned). An unplanned registration/visit means that it was not scheduled and could therefore be acute or sub-acute. Every stay at a ward resulted in a registration. This means that if an individual changed wards during the hospital stay there would be several registrations for the same in-hospital period. Healthcare utilisation for somatic care was identified based on the clinic to which the individual had visited or been admitted. All healthcare utilisation information was registered by staff at the different healthcare facilities at the time of the visit. NPR data used in the present study can be found in Table 1.

**Table 1:** Description of used variables in the National Patient Register

Inpatient care	Outpatient care	Used for:
Hospital/Clinic	Hospital/Clinic	Identifying somatic healthcare
Date of registration	Date of physician visit	Determining year of utilisation and the number of registrations/visits
Planned/unplanned registration	Planned/unplanned visit	Determining whether or not the registration/visit was planned
Length of stay	-	Length of stay for each registration

### Statistical analysis

Age cohorts at 5-year intervals were created for each study year. Differences between the ID and control groups were investigated with respect to four different outcomes: planned/unplanned inpatient registrations/outpatient physician visits. For each outcome, year, and age group, odds ratios (OR) with 95 % confidence intervals for at least one registration/visit were estimated using logistic regression (presented in the Additional file 1). To visualize patterns, ORs based on age groups with at least 100 participants were plotted for each year and outcome. In addition, repeated-measure analyses were performed, with ORs for differences between the ID and control groups calculated and plotted for the whole study period, regardless of year. To investigate ORs and age patterns within the ID group, repeated-measures analyses were conducted, using the largest group (aged 55–59 years) as the reference. The regression analyses were not controlled for any variables, as the groups were already age- and sex matched, and as no other background variables were available. Differences in number of visits were assessed by a Mann–Whitney *U*-test, as the data were severely skewed. However, as most medians and quartiles were equal for those with at least one registration/visit, means and standard deviations (SDs) are presented rather than medians and quartiles. Here, age groups were merged into 10-year intervals to allow presentation of the large amount of data. Only comparisons with at least five individuals in each group are presented. Changes between age groups were investigated with Chi-square tests for trends for nominal data, and Jonckheere–Terpstra tests for skewed ratio data.

All analyses were performed with IBM SPSS statistics for Window, Version 23 (IBM Corp, Armonk, NY, USA).

## Results

### Patterns of somatic healthcare utilization

**Fig. 1:**
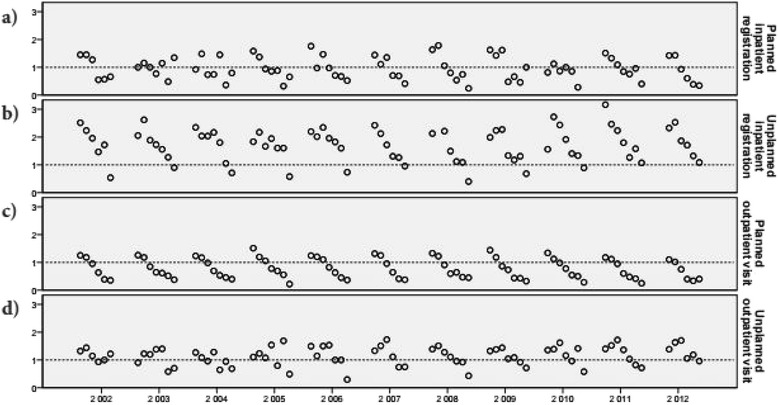
Odds ratios (intellectual disability group vs. control group) for those with ≥1 registration/visit for 2002–2012. Comparisons are made independently (2002–2012) for each year, for the intellectual disability group in relation to the control group, and for planned inpatient registrations (**a**), unplanned inpatient registrations (**b**), planned outpatient visits (**c**), and unplanned outpatient visits (**d**﻿﻿) respectively. Each circle represents one 5-year age group. Age groups are presented in order with the youngest age group to the left and the oldest to the right for each year. Only age groups with more than 100 individuals are presented here. All data are presented in the Additional file 1

For all types of healthcare utilization investigated (planned/unplanned in- and outpatient care), a decrease in utilization in the ID group compared with the control group was found with increasing age (Fig. 1a–d, details presented in the Additional file 1). Those in the younger age groups in the ID group were more likely to utilise healthcare than their peers in the control group, while the opposite was seen in the older age groups. This was most evident in planned outpatient visits (Fig. 1c). For unplanned inpatient visits (Fig. 1b), the ID group had a higher proportion of individuals with at least one registration in most age groups, with an OR below 1 only in the oldest age group.

**Fig. 2:**
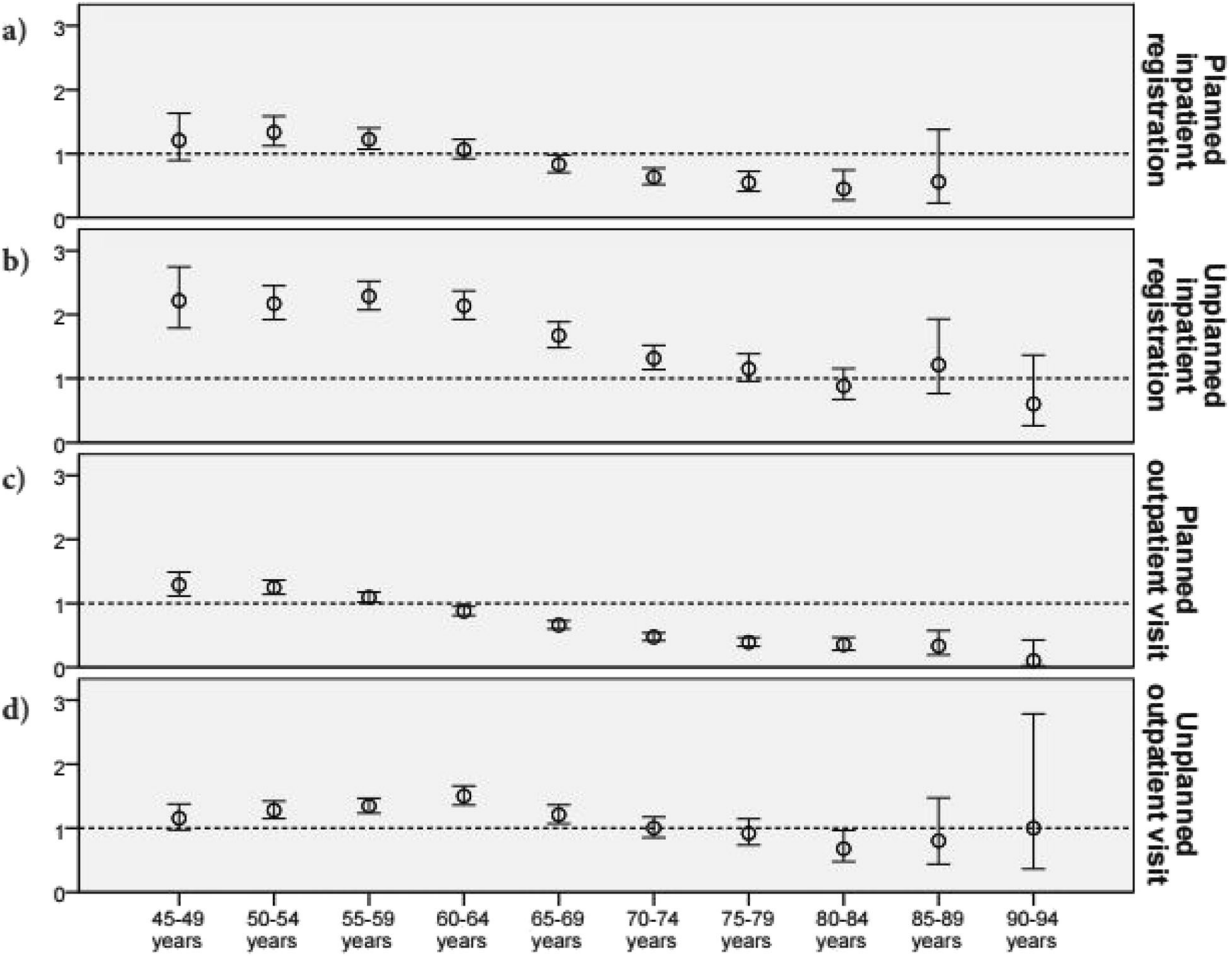
Odds ratios (intellectual disability group vs. control group) for those with ≥1 registration/visit for each age group. Repeated-measures analyses (independent of year) for the intellectual disability group in relation to the control group for each age group for planned inpatient registrations (**a**), unplanned inpatient registrations (**b**), planned outpatient visits (**c**), and unplanned outpatient visits (**d**﻿) respectively. Only age groups with more than 100 individuals are presented here. All data are presented in the Additional file 1

The same patterns are also seen in Fig. 2, where the ORs for each age group are presented independently of year (details presented in the Additional file 1). This pattern was obvious for planned inpatient registrations (Fig. 2a) and planned outpatient visits (Fig. 2c), with ORs above 1 in the younger age groups and below 1 in the older age groups. Unplanned inpatient registrations (Fig. 2b) showed a decreasing trend, but an OR below 1 only in the oldest age group.

**Fig. 3:**
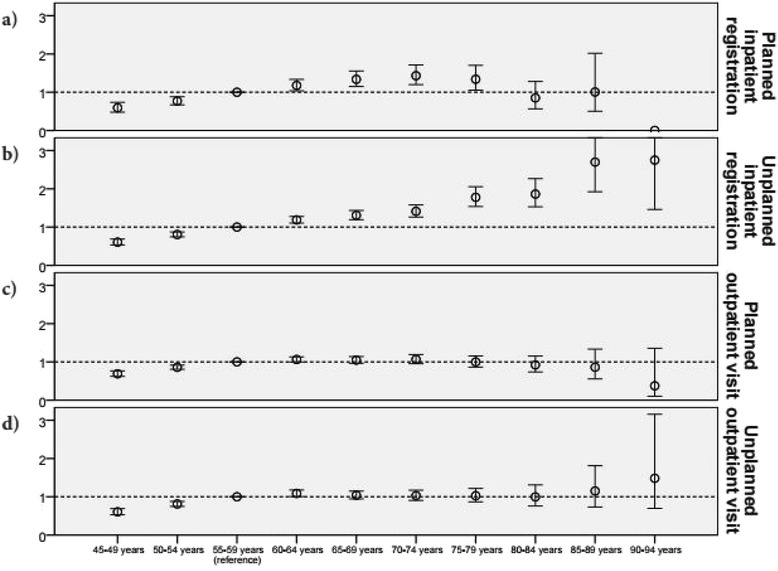
Odds ratios between age groups for those in the intellectual disability group with ≥1 registration/visit. Repeated-measures analyses (independent of year) for each age group in relation to the group aged 55–59 years (reference) ﻿for planned inpatient registrations (**a**), unplanned inpatient registrations (**b**), planned outpatient visits (**c**), and unplanned outpatient visits (**d**﻿) respectively. Only age groups with more than 100 individuals are presented here. All data are presented in the Additional file 1

When examining the age effect within the ID group, with the group aged 55–59 years as the reference group, Fig. 3 shows less clear patterns with increasing age (details presented in the Additional file 1). For planned inpatient registrations, there was an increase until the age 70–74 years, and thereafter a decline (in relation to the group aged 55–59 years) (Fig. 3a). For unplanned inpatient registrations, there was a clear pattern of increasing OR with age (Fig. 3b). For planned outpatient visits, there was a small increase until the age 70–74 years, followed by a decrease (Fig. 3c). The OR for unplanned outpatient visits also showed a small increase with age when considering all age groups. However, between the groups aged 55–59 years and 80–84 years, the OR was relatively stable (Fig. 3d).

### Planned somatic inpatient care and planned LOS for those with at least one registration

Among those with at least one planned inpatient registration, less visits were found in the ID group than in the control group for those aged 45–54 years (in 2002) and 55–64 years (in 2011) (Table 2). There were no linear trends for age in planned inpatient registrations. For planned LOS, the ID group had longer stays for those aged 45–54 years (in 2002), 55–64 years (in 2008), and 75–84 years (in 2010) (Table 2). Planned LOS increased with age in the ID group in 2005 and 2010, and in the control group in 2004, 2009, and 2011.

**Table 2:** Mean number of planned somatic inpatient registrations and length of stay among those with at least one registration

		Planned inpatient registrations						Planned LOS					
Year		Age 45-54	Age 55-64	Age 65-74	Age 75-84	Age 85-94	Trend *p*-value^b^	Age 45-54	Age 55-64	Age 65-74	Age 75-84	Age 85-94	Trend *p*-value^b^
2002	ID (SD)	**1.08 (0.31)**	1.28 (0.56)	1.07 (0.26)			0.087	**7.36 (19.71)**	7.40 (8.61)	3.67 (4.12)			0.170
	G (SD)	**1.19 (0.39)**	1.25 (0.51)	1.20 (0.41)			0.812	**6.24 (7.26)**	6.53 (14.32)	5.04 (4.26)			0.440
	*p*-value^a^	**0.035**	0.882	0.259				**0.040**	0.214	0.184			
2003	ID (SD)	1.16 (0.44)	1.19 (0.43)	1.21 (0.72)			0.627	8.56 (14.55)	5.29 (6.31)	6.83 (10.75)			0.737
	G (SD)	1.37 (1.32)	1.18 (0.52)	1.07 (0.37)			0.106	6.48 (15.02)	7.23 (20.10)	7.70 (13.64)			0.151
	*p*-value^a^	0.640	0.642	0.417				0.369	0.148	0.434			
2004	ID (SD)	1.24 (0.56)	1.10 (0.31)	1.22 (0.64)	1.00 (0.00)		0.178	8.90 (17.83)	5.01 (6.07)	7.89 (9.02)	8.20 (7.16)		0.282
	G (SD)	1.19 (0.53)	1.21 (0.59)	1.27 (0.65)	1.67 (1.63)		0.544	4.49 (5.66)	6.08 (9.56)	7.05 (8.81)	8.00 (9.19)		**0.023**
	*p*-value^a^	0.448	0.409	0.590	0.361			0.126	0.648	0.422	1.000		
2005	ID (SD)	1.25 (0.53)	1.15 (0.47)	1.21 (0.41)	1.00 (0.00)		0.280	4.37 (5.29)	5.67 (5.96)	9.17 (11.15)	5.14 (5.18)		**0.024**
	G (SD)	1.15 (0.47)	1.19 (0.61)	1.11 (0.39)	1.10 (0.32)		0.786	4.49 (4.29)	5.87 (15.65)	5.02 (4.94)	4.30 (2.58)		0.800
	*p*-value^a^	0.168	0.955	0.179	0.403			0.194	0.127	0.257	0.921		
2006	ID (SD)	1.23 (0.71)	1.26 (0.61)	1.51 (0.95)	1.00 (0.00)		0.267	6.07 (10.41)	8.37 (21.82)	7.17 (11.23)	3.30 (2.98)		0.255
	G (SD)	1.33 (0.72)	1.23 (0.65)	1.24 (0.48)	1.81 (1.80)		0.484	5.50 (6.23)	4.65 (7.53)	5.04 (6.55)	23.06 (56.96)		0.318
	*p*-value^a^	0.336	0.485	0.243	0.055			0.695	0.500	0.601	0.134		
2007	ID (SD)	1.30 (0.85)	1.20 (0.49)	1.29 (0.79)	1.20 (0.63)		0.991	8.98 (19.92)	5.94 (8.00)	6.10 (5.55)	5.80 (7.21)		0.056
	G (SD)	1.32 (0.94)	1.34 (0.80)	1.35 (0.68)	1.25 (0.72)		0.545	11.62 (37.18)	7.46 (10.68)	8.94 (11.64)	7.05 (17.63)		0.547
	*p*-value^a^	0.945	0.432	0.364	0.738			0.673	0.298	0.667	0.859		
2008	ID (SD)	1.13 (0.40)	1.28 (0.67)	1.11 (0.32)	1.14 (0.38)		0.998	4.91 (6.94)	**8.84 (15.49)**	6.09 (8.62)	6.14 (6.64)		0.772
	G (SD)	1.17 (0.47)	1.28 (0.71)	1.22 (0.52)	1.42 (0.51)		0.463	6.55 (10.53)	**5.23 (7.58)**	4.37 (4.54)	7.50 (7.93)		0.440
	*p*-value^a^	0.675	1.000	0.358	0.228			0.926	**0.043**	0.789	0.608		
2009	ID (SD)	1.20 (0.40)	1.22 (0.62)	1.24 (0.62)	1.19 (0.54)		0.513	8.33 (14.65)	7.22 (13.69)	7.80 (9.50)	8.06 (14.34)		0.737
	G (SD)	1.21 (0.79)	1.25 (0.56)	1.21 (0.63)	1.39 (0.88)		0.680	7.11 (17.56)	5.51 (7.05)	6.28 (7.65)	9.32 (12.56)		**0.037**
	*p*-value^a^	0.341	0.604	0.710	0.349			0.286	0.908	0.837	0.369		
2010	ID (SD)	1.17 (0.51)	1.13 (0.39)	1.19 (0.54)	1.33 (0.50)		0.322	6.17 (12.70)	6.47 (10.10)	6.36 (8.18)	**22.89 (20.82)**		**0.008**
	G (SD)	1.41 (0.73)	1.22 (0.52)	1.28 (0.70)	1.26 (0.45)		0.983	11.91 (21.98)	8.40 (22.56)	5.23 (6.13)	**6.82 (7.43)**		0.458
	*p*-value^a^	0.212	0.171	0.285	0.675			0.056	0.709	0.328	**0.009**		
2011	ID (SD)	1.08 (0.29)	**1.15 (0.54)**	1.27 (0.64)	1.17 (0.46)		0.111	3.00 (2.70)	5.62 (7.58)	7.17 (11.82)	6.87 (7.40)		0.211
	G (SD)	1.25 (0.46)	**1.30 (0.72)**	1.30 (0.63)	1.39 (0.97)		0.368	6.25 (5.12)	4.51 (4.97)	5.64 (6.83)	10.55 (11.48)		**0.023**
	*p*-value^a^	0.319	**0.019**	0.581	0.367			0.140	0.784	0.686	0.193		
2012	ID (SD)		1.21 (0.56)	1.23 (0.56)	1.15 (0.37)		0.767		6.83 (13.83)	5.68 (7.60)	4.50 (4.94)		0.716
	G (SD)		1.30 (0.69)	1.27 (0.59)	1.37 (1.00)		0.789		6.40 (14.15)	5.63 (5.60)	7.90 (10.35)		0.062
	p-value^a^		0.279	0.442	0.483				0.905	0.075	0.358		

^a^Mann–Whitney *U*-test, ^b^Jonckheere–Terpstra test for trend, Bold text = statistically significant, *LOS* length of stay, *ID* intellectual disability group, *G* general population sample, *SD* standard deviation

### Unplanned somatic inpatient care and unplanned LOS for those with at least one registration

Among those with unplanned inpatient registrations, some statistically significant higher values were seen in the ID group in the younger age groups (one in the group aged 45–54 years, four in the group aged 55–64 years, and one in the group aged 65–74 years) (Table 3). In the group aged 78–84 years, one statistically significant difference was found, with higher values in the control group. Increasing trends were seen only in the control group for three of the study years (2007, 2011, and 2012).

**Table 3:** Mean number of unplanned somatic inpatient registration and length of stay among those with at least one registration

		Unplanned inpatient registrations						Unplanned LOS					
Year		Age 45-54	Age 55-64	Age 65-74	Age 75-84	Age 85-94	Trend *p*-value^b^	Age 45-54	Age 55-64	Age 65-74	Age 75-84	Age 85-94	Trend *p*-value^b^
2002	ID (SD)	**1.59 (1.65)**	1.54 (1.28)	1.15 (0.41)	1.44 (0.73)		0.281	**5.94 (7.28)**	**8.14 (12.37)**	6.31 (7.69)	9.67 (9.21)		0.116
	G (SD)	**1.39 (1.28)**	1.37 (0.94)	1.27 (0.59)	1.38 (0.74)		0.528	**4.19 (7.14)**	**3.60 (4.29)**	5.86 (9.68)	5.63 (7.63)		**0.035**
	*p*-value^a^	**0.028**	0.178	0.281	0.764			**<0.001**	**<0.001**	0.482	0.209		
2003	ID (SD)	1.47 (1.25)	1.45 (1.04)	1.62 (1.30)	1.36 (0.92)		0.262	6.08 (9.27)	**7.20 (11.05)**	**8.21 (11.16)**	3.18 (1.47)		**0.001**
	G (SD)	1.33 (0.92)	1.38 (0.93)	1.29 (0.64)	1.08 (0.28)		0.812	4.09 (4.58)	**4.83 (6.41)**	**4.78 (6.52)**	3.38 (2.90)		0.972
	*p*-value^a^	0.435	0.360	0.259	0.420			0.229	**0.001**	**0.005**	0.930		
2004	ID (SD)	1.66 (1.44)	1.46 (1.11)	**1.41 (0.84)**	1.19 (0.40)		0.053	**7.44 (10.18)**	**7.44 (11.24)**	9.41 (15.55)	8.00 (9.35)		0.200
	G (SD)	1.53 (1.46)	1.27 (0.71)	**1.84 (1.12)**	1.14 (0.47)		0.470	**5.33 (11.25)**	**3.72 (4.53)**	7.31 (10.03)	5.00 (4.59)		**0.038**
	*p*-value^a^	0.287	0.179	**0.004**	0.435			**<0.001**	**<0.001**	0.356	0.341		
2005	ID (SD)	1.57 (1.57)	1.67 (1.58)	1.38 (0.84)	1.55 (1.10)		0.950	6.96 (14.94)	**7.72 (12.11)**	**7.34 (7.41)**	6.55 (7.66)		**0.007**
	G (SD)	1.37 (0.78)	1.32 (0.70)	1.78 (1.65)	1.43 (0.79)		0.459	4.73 (6.55)	**4.24 (6.82)**	**5.48 (7.73)**	6.75 (8.16)		0.406
	*p*-value^a^	0.664	0.073	0.271	0.768			0.156	**<0.001**	**0.014**	0.627		
2006	ID (SD)	1.38 (0.91)	1.53 (1.46)	1.39 (1.00)	1.26 (0.53)		0.794	5.80 (8.94)	**5.92 (8.67)**	**6.87 (8.35)**	6.70 (5.86)		**0.002**
	G (SD)	1.36 (0.83)	1.46 (1.10)	1.37 (1.07)	1.26 (0.68)		0.685	6.23 (13.23)	**5.15 (10.18)**	**5.17 (8.09)**	7.16 (9.53)		0.390
	*p*-value^a^	0.886	0.585	0.898	0.623			0.485	**0.023**	**0.005**	0.319		
2007	ID (SD)	1.48 (0.99)	1.63 (1.50)	1.41 (0.91)	**1.30 (0.53)**		0.414	**7.31 (11.77)**	**8.33 (10.24)**	7.95 (9.79)	6.00 (4.22)		**0.045**
	G (SD)	1.44 (1.12)	1.51 (1.51)	1.42 (0.82)	**1.72 (0.92)**		**0.010**	**4.27 (8.44)**	**5.54 (12.50)**	6.35 (7.80)	6.62 (6.26)		**<0.001**
	*p*-value^a^	0.315	0.084	0.579	**0.029**			**0.001**	**<0.001**	0.095	0.816		
2008	ID (SD)	1.47 (1.02)	1.65 (1.43)	1.37 (0.82)	1.45 (0.80)		0.651	6.63 (11.33)	**8.10 (10.88)**	**6.55 (6.09)**	6.11 (5.13)		**0.041**
	G (SD)	1.51 (1.27)	1.45 (0.93)	1.54 (1.34)	1.41 (0.89)		0.736	6.02 (9.74)	**5.78 (8.63)**	**6.64 (10.76)**	6.67 (8.25)		0.280
	*p*-value^a^	0.712	0.171	0.902	0.365			0.212	**<0.001**	**0.003**	0.311		
2009	ID (SD)	1.70 (1.77)	**1.61 (1.19)**	1.71 (1.27)	1.64 (1.40)		0.672	**8.55 (17.95)**	**8.33 (12.55)**	8.99 (11.65)	8.59 (8.95)		0.093
	G (SD)	1.64 (1.39)	**1.38 (0.86)**	1.65 (1.16)	1.68 (1.33)		0.053	**5.09 (7.98)**	**4.66 (6.86)**	7.24 (8.77)	8.29 (8.44)		**<0.001**
	*p*-value^a^	0.873	**0.011**	0.989	0.560			**0.003**	**<0.001**	0.267	0.764		
2010	ID (SD)	1.68 (1.65)	**1.63 (1.20)**	1.55 (1.26)	1.59 (0.93)	1.58 (0.67)	0.698	7.08 (10.96)	**7.15 (9.81)**	**7.83 (9.72)**	7.90 (9.16)	8.75 (7.71)	**0.009**
	G (SD)	1.48 (1.04)	**1.44 (1.00)**	1.57 (1.19)	1.55 (1.09)	1.33 (0.90)	0.279	7.58 (14.44)	**4.67 (6.40)**	**7.12 (10.89)**	8.75 (9.95)	8.87 (7.82)	**<0.001**
	*p*-value^a^	0.634	**0.027**	0.526	0.374	0.095		0.120	**<0.001**	**0.045**	0.868	1.000	
2011	ID (SD)	1.56 (1.41)	**1.71 (1.23)**	1.62 (1.33)	1.75 (1.40)	1.82 (1.07)	0.996	5.36 (7.72)	**8.19 (10.89)**	8.03 (9.89)	8.58 (9.89)	6.47 (5.79)	**0.016**
	G (SD)	1.09 (0.29)	**1.46 (1.12)**	1.65 (1.47)	1.79 (1.30)	1.73 (1.22)	**<0.001**	3.55 (5.46)	**5.41 (12.11)**	7.69 (11.68)	11.64 (14.76)	7.47 (4.64)	**<0.001**
	*p*-value^a^	0.076	**<0.001**	0.729	0.737	0.674		0.203	**<0.001**	0.090	0.207	0.471	
2012	ID (SD)		**1.70 (1.37)**	1.79 (1.40)	1.66 (1.09)	1.71 (1.12)	0.276		**7.83 (11.50)**	**8.57 (10.07)**	11.11 (14.91)	8.13 (6.90)	**<0.001**
	G (SD)		**1.64 (1.74)**	1.69 (1.27)	1.71 (1.32)	2.04 (1.35)	**0.021**		**6.19 (10.06)**	**8.07 (11.05)**	9.82 (11.54)	16.75 (17.62)	**<0.001**
	*p*-value^a^		**0.049**	0.137	0.886	0.299			**0.005**	**0.006**	0.299	0.061	

^a^Mann–Whitney *U*-test, ^b^Jonckheere–Terpstra test for trend, Bold text = statistically significant, *LOS* length of stay, *ID* intellectual disability group, *G* general population sample, *SD* standard deviation

More statistically significant differences were seen when investigating unplanned inpatient LOS. For the three youngest age groups, the ID group had statistically significant higher means in 21 of 32 comparisons (stratified by year) (Table 3). No statistically significant differences were found in the two oldest age groups. There was a statistically significantly increasing trend for age in eight of the 11 study years in the ID group, and in seven of the 11 study years in the control group.

### Planned physician visits in somatic outpatient care for those with at least one visit

When looking at the number of planned visits to physicians in outpatient care among those with at least one planned outpatient visit, there were 16 significant differences in total (four in the group aged 45–54 years, four in the group aged 55–64 years, six in the group aged 65–74 years, and two in the group aged 75–84 years); all of which showed lower values in the ID group compared with the control group (Table 4). There was an increasing trend with age in the control group in four of the 11 study years.

**Table 4:** Mean number of physician visits in somatic outpatient specialist care among those with at least one visit

		Planned outpatient visits to physician						Unplanned outpatient visits to physician					
Year		Age 45-54	Age 55-64	Age 65-74	Age 75-84	Age 85-94	Trend *p*-value^b^	Age 45-54	Age 55-64	Age 65-74	Age 75-84	Age 85-94	Trend *p*-value^b^
2002	ID (SD)	**1.59 (1.11)**	1.74 (1.95)	1.98 (2.10)	1.13 (0.35)		0.825	**1.71 (1.83)**	1.79 (2.94)	1.63 (2.47)			0.404
	G (SD)	**1.85 (1.47)**	1.70 (1.23)	1.82 (1.25)	1.83 (1.00)		0.529	**1.19 (0.49)**	1.41 (0.96)	1.23 (0.55)			0.069
	*p*-value^a^	**0.017**	0.101	0.574	0.057			**<0.001**	0.309	0.675			
2003	ID (SD)	1.62 (1.44)	1.72 (2.05)	1.68 (1.19)	1.41 (0.80)		0.318	**1.78 (2.24)**	**1.72 (2.64)**	1.24 (0.43)	1.20 (0.45)		0.945
	G (SD)	1.72 (1.72)	1.78 (1.40)	1.73 (1.34)	2.00 (1.43)		0.290	**1.25 (0.64)**	**1.22 (0.73)**	1.32 (0.64)	1.14 (0.38)		0.946
	*p*-value^a^	0.093	0.076	0.984	0.147			**0.014**	**0.001**	0.956	0.802		
2004	ID (SD)	1.79 (1.85)	1.73 (1.54)	1.61 (1.00)	1.50 (0.99)		0.738	**1.55 (1.32)**	**1.70 (3.41)**	1.37 (0.82)	1.38 (0.74)		0.625
	G (SD)	1.80 (1.96)	1.89 (1.96)	1.86 (1.42)	2.06 (1.76)		0.022	**1.25 (0.58)**	**1.22 (0.70)**	1.42 (0.91)	1.45 (0.52)		0.315
	*p*-value^a^	0.871	0.254	0.105	0.191			**0.019**	**0.010**	0.962	0.527		
2005	ID (SD)	1.68 (1.33)	1.81 (1.69)	1.71 (1.40)	1.72 (1.27)		0.835	1.42 (1.01)	1.62 (1.97)	1.34 (0.75)	1.50 (0.84)		0.843
	G (SD)	1.80 (2.14)	1.89 (1.71)	1.81 (1.38)	1.80 (1.09)		0.080	1.26 (0.72)	1.34 (0.81)	1.18 (0.51)	1.11 (0.33)		0.901
	*p*-value^a^	0.885	0.254	0.131	0.590			0.101	0.586	0.219	0.273		
2006	ID (SD)	1.77 (1.50)	1.95 (2.56)	**1.70 (1.47)**	1.78 (1.40)		0.984	**1.40 (0.86)**	1.54 (1.67)	1.42 (0.85)	1.50 (0.84)		0.948
	G (SD)	1.78 (1.88)	2.16 (3.21)	**2.16 (2.47)**	2.00 (1.37)		**0.006**	**1.21 (0.69)**	1.28 (0.65)	1.35 (0.97)	1.32 (0.58)		0.070
	*p*-value^a^	0.441	0.927	**0.049**	0.195			**0.009**	0.590	0.473	0.657		
2007	ID (SD)	**1.64 (1.32)**	1.76 (1.95)	**1.63 (1.08)**	1.50 (0.77)		0.342	1.45 (1.55)	1.49 (1.38)	1.32 (0.93)	1.28 (0.46)		0.297
	G (SD)	**1.85 (1.57)**	1.86 (1.87)	**2.19 (2.80)**	2.59 (4.07)		0.134	1.26 (0.73)	1.30 (0.85)	1.21 (0.49)	1.33 (0.55)		0.353
	*p*-value^a^	**0.032**	0.104	**0.050**	0.092			0.053	0.076	0.967	0.819		
2008	ID (SD)	**1.49 (0.97)**	1.83 (2.03)	**1.52 (0.90)**	1.75 (1.18)		0.203	1.42 (1.01)	1.51 (1.50)	1.34 (0.72)	1.25 (0.51)		0.746
	G (SD)	**2.36 (3.85)**	1.89 (2.37)	**2.00 (2.22)**	2.25 (3.32)		0.415	1.30 (0.70)	1.31 (0.90)	1.35 (0.74)	1.28 (0.60)		0.722
	*p*-value^a^	**<0.001**	0.571	**0.008**	0.318			0.815	0.132	0.877	0.930		
2009	ID (SD)	**1.51 (0.87)**	**1.67 (1.15)**	**1.71 (1.73)**	1.72 (1.09)		0.453	1.59 (1.74)	1.31 (0.81)	1.47 (1.11)	1.36 (0.59)		0.649
	G (SD)	**1.87 (1.40)**	**2.03 (2.32)**	**1.97 (1.57)**	2.23 (1.94)		**0.024**	1.27 (0.78)	1.47 (2.10)	1.45 (0.78)	1.45 (1.15)		**0.034**
	*p*-value^a^	**0.047**	**0.027**	**0.001**	0.062			0.080	0.238	0.495	0.487		
2010	ID (SD)	1.57 (1.06)	**1.72 (1.25)**	1.88 (2.06)	**1.59 (0.89)**	1.80 (1.03)	0.088	1.45 (1.45)	1.46 (1.19)	1.27 (0.60)	1.32 (0.73)	1.20 (0.45)	0.246
	G (SD)	2.21 (3.38)	**2.20 (3.14)**	2.11 (2.15)	**2.41 (4.08)**	1.71 (1.31)	0.107	1.24 (0.60)	1.28 (0.67)	1.32 (0.80)	1.26 (0.68)	1.00 (0.00)	0.962
	*p*-value^a^	0.116	**0.006**	0.050	**0.035**	0.449		0.173	0.283	0.938	0.753	0.273	
2011	ID (SD)	1.79 (1.12)	**1.85 (1.69)**	**1.89 (2.13)**	1.73 (1.20)	1.71 (1.11)	0.766	1.36 (0.98)	**1.45 (1.03)**	1.29 (0.76)	**1.45 (0.70)**	1.00 (0.00)	0.586
	G (SD)	1.76 (1.50)	**2.25 (3.41)**	**2.33 (2.82)**	2.13 (1.74)	1.86 (0.95)	**0.002**	1.31 (0.52)	**1.28 (0.82)**	1.44 (0.94)	**1.29 (0.87)**	1.33 (0.71)	0.564
	*p*-value^a^	0.172	**0.049**	**0.001**	0.063	0.544		0.489	**0.006**	0.136	**0.025**	0.198	
2012	ID (SD)		**1.90 (2.40)**	**1.83 (1.58)**	**1.59 (1.16)**	1.63 (0.81)	0.233		1.52 (1.42)	**1.45 (1.04)**	1.20 (0.46)	1.55 (0.82)	0.821
	G (SD)		**2.01 (1.78)**	**2.55 (3.20)**	**2.44 (2.11)**	1.72 (1.09)	**<0.001**		1.32 (0.74)	**1.29 (0.71)**	1.27 (0.57)	1.50 (0.89)	0.695
	*p*-value^a^		**0.005**	**<0.001**	**<0.001**	0.859			0.389	**0.043**	0.574	0.790	

^a^Mann–Whitney *U*-test, ^b^Jonckheere–Terpstra test for trend, Bold text = statistically significant, *ID* = Intellectual disability group, *G* general population sample, *SD* standard deviation

### Unplanned physician visits in somatic outpatient care for those with at least one visit

For those with at least one unplanned visit to physician in outpatient care, statistically significant higher values were found in the ID group compared with the control group in the youngest age group (45–54 years) in four of the 11 study years, the group aged 55–64 years in three of the study years, and for one of the study years each in the groups aged 65–74 years and 75–84 years (Table 4). For unplanned physician visits in outpatient care, there was only one statistically significant positive trend with age in the control group in 2009.

## Discussion

The results of the present study showed that people with ID have a different healthcare utilisation pattern with a decreasing risk of healthcare utilisation with age compared with the general population.

In general, younger people with ID utilise more unplanned and planned healthcare in terms of inpatient registrations and outpatient care visits compared with the same age groups in the general population (Fig. 1). This is not surprising, as people with ID have higher rates of many diseases [3–8], many of which may cause planned or unplanned physician visits for outpatient care or hospital admissions. The more complex disease patterns in people with ID may also be a reason for the longer LOS in those with at least one inpatient registration than in the general population (Table 3).

In the oldest age groups, fewer people in the ID group utilised healthcare compared with the general population. All four investigated forms of healthcare utilisation showed a pattern of decreasing ORs, with ORs below 1 in the oldest age groups. This pattern is consistent with a recently published study from Norway [41] that showed people with ID were more frequently hospitalized at a younger age and less frequently at old age. Low levels of unplanned care might occur if, when reaching older age, people with ID were well monitored by the healthcare system and therefore had their healthcare needs met elsewhere, in particular in primary care settings. Not all parts of the health system were included in the present study and thus, this could not be controlled for. Nevertheless, if people with ID were sufficiently monitored, this should reasonably lead to high levels of planned healthcare, especially considering their reduced health in comparison with the general population, as shown in a previous study using the same sample as in the present study [3]. However, this is contradicted by our finding that people with ID in the oldest age groups had lower proportions of planned in- and outpatient care than the general population. People with high utilisation in one provider have been demonstrated to also have high utilisation in other areas [51]. It has been reported that people with ID with more than 24 outpatient visits had 2.81 greater odds of being hospitalized [33], meaning it is unlikely that those with low healthcare utilisation in the present study would have high utilisation of other services (e.g., primary care). Regardless of whether or not health needs are met elsewhere in the health system, it is reasonable to expect that older people with ID, who are known to have more diseases, will have higher rates of healthcare use, or at least rates that are not lower than in the general population.

In addition, the results of the present study showed that for those with at least one planned visit to physician in outpatient care, the ID group had fewer visits compared with the control group (Table 4). The reason why people with ID have fewer registrations of planned healthcare needs further study. Some studies have suggested that people with ID may have difficulty accessing the health system [14, 52], with lower rates of preventive healthcare such as breast and cervical cancer screening [4, 10] and influenza vaccination [10, 53]. The health system’s different treatment of people with ID compared with the general population is supported by several reviews ﻿[54–57]. These reviews identified experiences of people with ID, parents, carers and/or healthcare staff of barriers that prevent access to quality healthcare. Reported barriers include experiencing the healthcare/hospital as a fearful encounter, negative attitudes and discrimination, lack of knowledge and formal training among staff, communication difficulties, and the diagnostic uncertainty that may be present when the person with ID has problems in verbal expressions of pain, anxiety, and distress [54–57].

Another possible explanation for our results is that in people with ID, the oldest age groups are healthier than their younger peers. This is somewhat contradicted by the in-group comparison of the ID group (Fig. 3) that revealed an increase of unplanned healthcare utilisation with age. Although it may seem unlikely that people with ID should become healthier with increasing age, this may occur if there was a selection of healthier people in the oldest age groups; that is, if those with severe and profound ID were less likely to survive into older age. This may result in higher proportions of people with mild or moderate ID in the oldest age groups compared with the younger age groups. However, even if there is such an overrepresentation these would be expected to suffer from age-related diagnoses and thus, rates of healthcare utilisation, to the same extent as their peers in the general population. Nonetheless, in the present study, we had no information about the severity of ID, which might have resulted in lower healthcare utilisation in the older age groups, and the results should therefore be interpreted with some caution.

Some limitations are mentioned above (lack of primary care data, the risk of a cohort effect, and not being able to discriminate levels of ID severity). In addition, only two variables was used when matching the control group (year of birth and sex) which might have increased the risk of selection bias and reduced internal validity. For example, the sample was not matched on place of residence, meaning that the ID and control group participants might have lived in different parts of the country with different distances to healthcare facilities, which might in turn have affected access to and utilisation of healthcare. As no information about place of residence for the total sample was available we do not know whether this caused an under- or overestimation in the general population sample. Socioeconomic variables were not considered as matching variables. There are studies reporting that people with ID have lower socioeconomic status (SES) than the general population [42, 58] and that poor SES is associated with lower access of healthcare in many countries [31, 59], including Sweden [45, 60, 61]. However, SES may be a link in the casual chain between ID and utilisation of healthcare and thus, controlling for these variables would also remove potential effects of the ID itself. There are also studies that have been unable to find associations between SES and healthcare utilisation [30, 32]. People with ID are predisposed to several conditions, such as lower SES, and the aim of this study was not to explore explanatory factors such as a certain condition or diagnosis, but to explore how the group of people with ID utilised healthcare utilisation in relation to the general population. In addition, the healthcare system in Sweden is mainly funded by taxes, i.e., all people regardless of income level are supposed to have equal access to healthcare. An increased number of matching variables would also make it more difficult to make an unbiased random selection of the general population sample. The results in this study are due to ID, to variables closely related to ID, or to variables that are consequences of ID and interpretations should be made with this in mind.

A person may have several registrations during one hospital stay (defined as one date for admission and one date for discharge), where some registrations could be planned and others unplanned. When the registrations were merged into hospital stay it was not possible to determine whether the stay was planned or unplanned. This means that the numbers in Table 3 are higher than the actual number of hospital stays. When comparing the total number of registrations and number of hospital stays, the number of registrations was about 5 % higher than hospital stays. Comparisons with other studies using hospital stay as an outcome should be made with this in mind.

In this study, the results of 398 statistical analyses are presented. Such a high number of tests may be a threat to statistical validity, as it increases the risk of mass significance (i.e., type I error) [62] with approximately a 5 % likelihood (26 tests) of incorrectly rejecting a null hypothesis. To reduce the risk of type I errors, conclusions are drawn on the patterns of the results rather than single *p*-values.

A strength of this study was that the registrations in the NPR are connected to the reimbursement system in Sweden, and the coverage is therefore generally high. For inpatient care, the coverage is almost 100 %, but coverage of outpatient care is lower, about 80 % [63]. The main reason for this lower number is a greater proportion of private providers for outpatient care compared with inpatient care, and more data from these are missing than data from public providers (coverage almost 100 %) [63]. Ludvigsson and colleagues [64] have investigated the validity of inpatient data in the Swedish NPR. They performed a review of 132 papers [64] with focus on registrations of diagnoses. The positive predictive value differed between diagnoses, but was in general between 85–95 %, and they concluded that the validity was high for most diagnoses [64].

The ID group in the present study were identified through a public national register and comprised a large group of people who were registered with ID during 2012 and who, at that time, were aged 55 years or older. A matched control group was also included, with both groups followed retrospectively for 11 years and included a very large set of data from the NPR. To the best of our knowledge, there is no other study investigating older people with ID covering such a large sample and over such a long time period. Altogether, a sample covering all people who received support and social services under the Act concerning Support and Service for Persons with Certain Functional Impairments [47], the matched general population sample, and the high validity of the public mandatory NPR, increases the external validity, meaning that these results may be generalized to similar contexts.

## Conclusions

The ID group had, in the youngest age groups, higher proportions of planned and unplanned in- and outpatient care than the control group. In the oldest age groups, the ID group had lower or similar proportions of unplanned care and lower proportions of planned care than the general population. It is likely that people with ID have reduced access to healthcare, because of their predisposition to having a low SES, which in turn is related to lower access. More research is needed to determine underlying reasons for this unique healthcare utilisation pattern.

## Additional file

Additional file 1: Data concerning differences in proportions with at least one visit registration with respect to four different outcomes: planned/unplanned inpatient registrations/outpatient physician visits(year, and age group, odds ratios (OR) with 95 % confidence intervals) and data concerning differences in number of visits, registrations and LOS for individuals with at least one registration/visit (mean, standarddeviations, *p*-values for Mann–Whitney U-tests, and Jonckheere–Terpstra tests for trend). (XLSX 53 kb)

## Abbreviations

ID: Intellectual disabilities
LOS: Length of stay
NPR: National Patient Register
OR: Odds ratio
SES: Socioeconomic status
